# Infectious Bursal Disease Virus Influences the Transcription of Chicken γ_c_ and γ_c_ Family Cytokines during Infection

**DOI:** 10.1371/journal.pone.0084503

**Published:** 2014-01-09

**Authors:** Sanying Wang, Qiaoyang Teng, Lu Jia, Xiaoyuan Sun, Yongping Wu, Jiyong Zhou

**Affiliations:** 1 Key Laboratory of Animal Virology of Ministry of Agriculture, Zhejiang University, Hangzhou, Zhejiang, People's Republic of China; 2 Collaborative Innovation Center for Diagnosis and Treatment of Infectious Diseases, Zhejiang University, Hangzhou, Zhejiang, People's Republic of China; 3 College of Animal Sciences and Technology, Zhejiang A&F University, Lin'an, Zhejiang, People's Republic of China; University of Hong Kong, China

## Abstract

Infectious bursal disease virus (IBDV) infection causes immunodeficiency in chickens. To understand cell-mediated immunity during IBDV infection, this study perform a detailed analysis of chicken γ_c_ chain (chCD132) and γ_c_ family cytokines, including interleukins 2, 4, 7, 9, and 15. The mouse anti-chCD132 monoclonal antibody (mAb) was first generated by the *E.coli*-expressed γ_c_ protein. Immunofluorescence assay further showed that γ_c_ was a protein located with the anti-chCD132 mAb on the surface of chicken's splenic mononuclear cells. Real-time quantitative RT-PCR revealed that the chCD132 mRNA transcript was persistently downregulated in embryo fibroblasts, spleen and thymus of chickens infected with IBDV. Correspondingly during IBDV infection, the transcription of five γ_c_ family cytokines was downregulated in the thymus and presented an imbalance in the spleen. Fluorescence-activated cell sorting analyses also indicated that the percentage of CD132^+^CD8^+^ T cells linearly decreased in the bursa of IBDV-infected chickens. These results confirmed that IBDV infection disturbed the *in vivo* balance of CD132 and γ_c_ family cytokine expression and that IBDV-induced immunodeficiency involved cellular networks related to the γ_c_ family.

## Introduction

Gamma c chain (γ_c_ or CD132) is identified as a cellular receptor responsible for the high affinity and signal transduction of IL-2 and is a shared cellular receptor of type I cytokines including IL-2, -4, -7, -9, -15, and -21 [1]. ILs sharing CD132 are named γ_c_ family cytokines. CD132 is expressed as a transmembrane glycoprotein on CD4^+^ and CD8^+^ T cells, B cells, NK cells, monocytes/macrophages, neutrophils, and granulocytes, but non-lymphocytes, such as keratinocytes and human gingival fibroblasts, also express CD132.

Structures of CD132 molecules with γ_c_ family cytokines have also been formulated [1]. CD132 is involved in either spontaneous or GH-induced cell cycle progression and regulates lymphocyte development and proliferation [2], [3]. Chicken CD132 (chCD132), the only CD132 molecule identified in domestic fowl, is transcribed in the spleen, thymus, and bursa of Fabricius (BF). Recently, we determined the structure of the chCD132 functional domain bound to chicken interleukin (chIL)-2 [4].

Infectious bursal disease virus (IBDV) is a member of the *Birnaviridae* family, and mainly replicates in the bursa of Fabricius (BF) of chickens. Replication of IBDV in the bursa is accompanied by an influx of T cells. The marked influx of T cells into the infected bursa indicates that cell-mediated immunity plays important roles in the clearance of virus particles. The T cells in the bursa of chickens infected by virus are activated, with up-regulated expression of a number of cytokine genes, such as IL-1b, IL-6, IFN-g, IL-2 and chIFN-γ. The change in the level of cytokine expression is closely associated with organizational destruction, inflammation and apoptosis. Further, extrabursal replication and persistence of the virus in vivo may determine the extent to which the cellular immune systems gets stimulated. IBDV induces an immunosuppressive response in chickens, which manifests as a necrosis of B lymphoid cells in the BF, a decrease in macrophages, a chIFN-γ over production by T lymphocytes and a subsequent reduction in the ability to respond to secondary infections. Recently, Liu *et al* [5] reported that IFN-γ, IL-2, IL-12P40, IL-4, IL-5, IL-10 and IL-13 are strongly induced during IBDV infection. However, the action of ILs and their cellular receptors in IBDV pathogenesis remains unknown. Here, we generated the mAb to chicken CD132 (chCD132) and analyzed the dynamic profiles of CD132 and γ_c_ family cytokines (γ_c_ cytokines) in IBDV-infected chickens. Our data confirmed that IBDV infection influences on the expression of CD132 and γ_c_ cytokines.

## Results

### Generation and Identification of the mAb to *chCD132*

**Figure 1:**
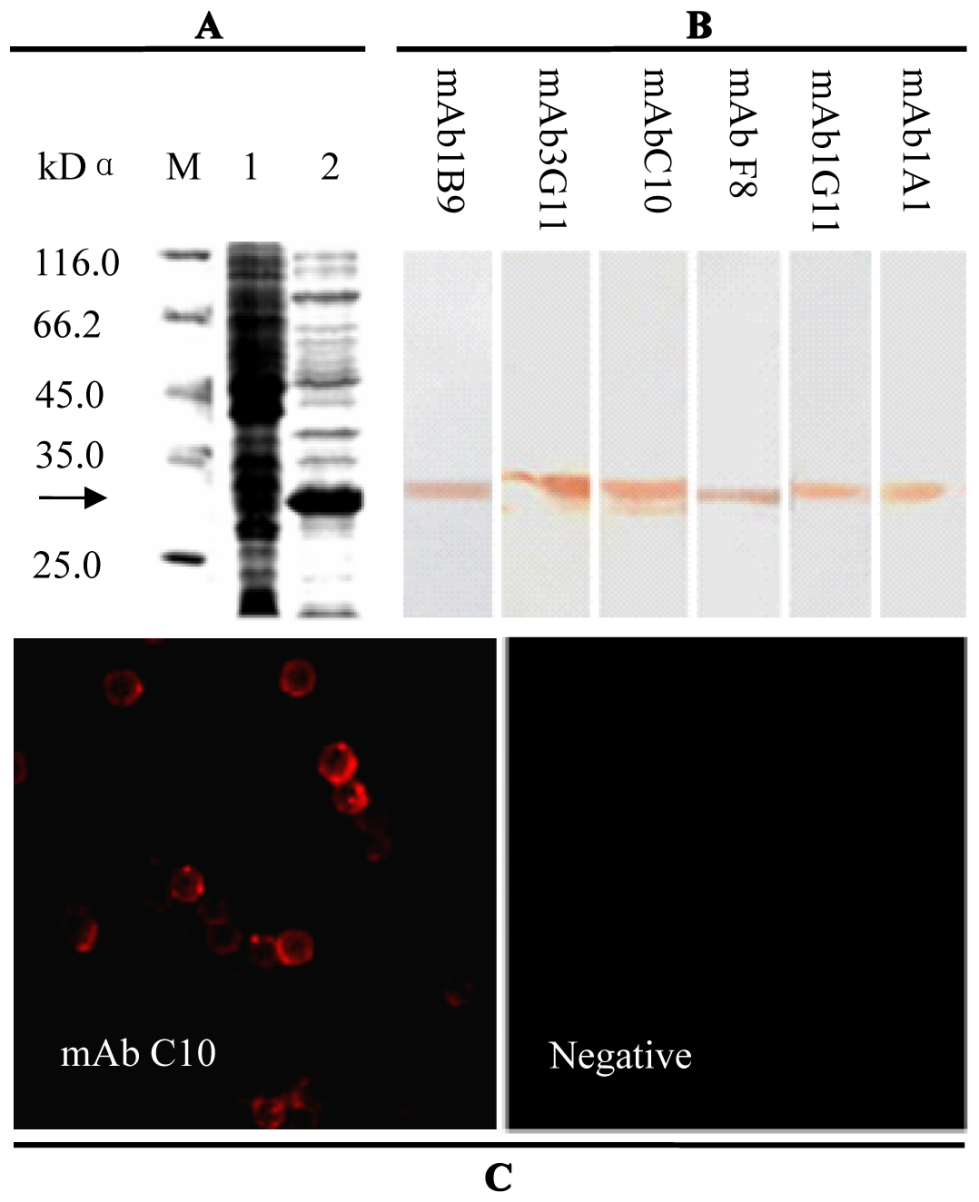
Identification of anti-chCD132 mAb bound to cellular CD132 on the SMC surface. (A) SDS-PAGE analysis of *Escherichia coli*-expressed chCD132; M, molecular weight marker; lane 1, bacterial lysates of *E. coli* BL21 (DE3) transformed with pET28a; lane 2, bacterial lysates containing rchCD132. (B) Western blot analysis of rchCD132 recognized by 6 anti-chCD132 mAbs. (C) anti-chCD132 mAb C10 recognized by chCD132 expressed on the SMC surface using indirect immunofluorescencestaining (×10).

The sequencing results showed that the *chCD132* cDNA sequence (GenBank Accession NO. D1852357) is 1047 bp in length and encodes a 327 amino acid (aa) polypeptide after truncation of the 21 aa signal peptide at the N-terminus. The *chCD132* cDNA was cloned into the pET28a plasmid. The resulting plasmid was transformed into the *E. coli* strain BL21 (DE3) for chCD132 expression, and rchCD132 (recombinant chCD132) with a 6×-His tag was optimally expressed in *E. coli* as insoluble inclusions after induction by 0.5 mM IPTG. As shown in Fig. 1A, the molecular weight of rchCD132 was approximately 28 kDa, according to SDS-PAGE results. Subsequently, rchCD132 protein was further purified by using a nickel column under denaturing conditions. SPF BALB/c mice were immunized subcutaneously with purified rchCD132, and 6 hybridoma cell lines secreting anti-chCD132 antibodies were established by the clone technique of limiting dilution. Western blot assays demonstrated that all 6 anti-chCD132 mAbs bound strongly to the rchCD132 protein expressed in *E. coli* (Fig. 1B); however, one (mAb C10) of the 6 mAbs exhibited the binding affinity similar to chCD132 protein expressed on the con A-stimulated SMC (Fig. 1C), indicating that the C10 mAb binds to cellular chCD132 located on the surface of SMC.

### Transcription and Expression of Gene chCD132 in IBDV-infected CEF

**Figure 2:**
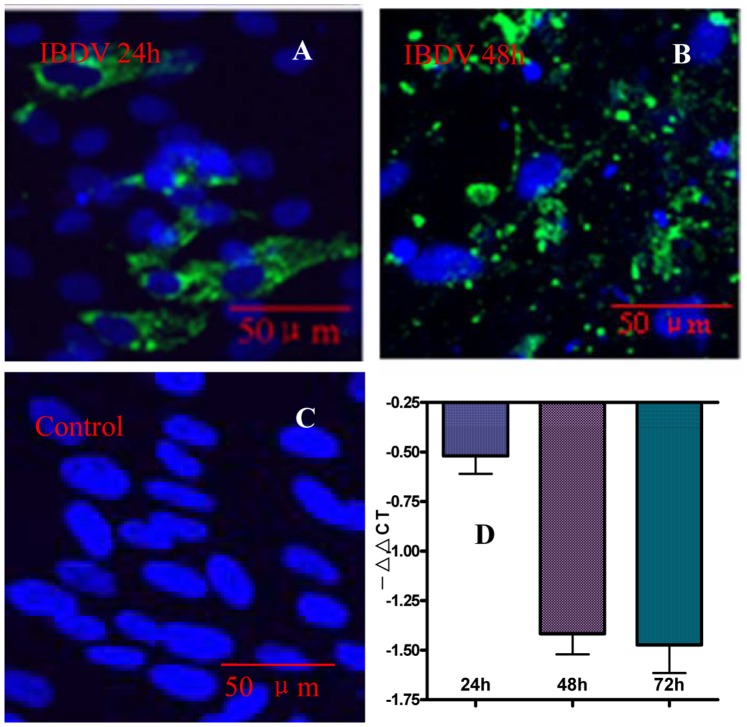
The mRNA abundance and protein expression of chCD132 on an IBDV-infected CEF monolayer. CEFs were infected with IBDV a 100 TCID dose of the eNB virus. (A)–(C) Double-stained immunofluorescence images with anti-chCD132 mAb (red) and chicken serum (green) to IBDV under laser confocal microscopy. (A), (B) and (C) There is no chCD132 (red) expression in IBDV- and mock-infected CEF. (D) Transcription kinetics of chCD132 analyzed by qRT-PCR. Samples were normalized with the β-actin gene as a control and uninfected CEF at each time point as a reference. Each experiment was conducted in triplicate. Values are expressed as −ΔΔCT ± SD.

The chCD132 expressed in IBDV-infected CEF was examined. As shown in Fig. 2A, 2B and 2C, chCD132 was not detected by the anti-chCD132 mAb C10 in the IBDV-inoculated and mock-infected CEF monolayer at 24 hpi and 48 hpi. These data demonstrate that chCD132 expression is not detected at a detectable protein level in uninfected and IBDV-infected CEF. To further analyze chCD132 changes on the transcriptional level, the *chCD132* transcript of the CEF monolayer with and without IBDV infection were analyzed at 24, 48, and 72 hpi by qRT-PCR. Data in Fig. 2D shows that during virus infection, compared with the mock-infected CEF monolayer, the γ_c_ mRNA level was persistently downregulated in the IBDV-infected CEF (p<0.05), indicating that γ_c_ mRNA transcription was inhibited during IBDV infection.

### In vivo Inhibition of chCD132 Expression during IBDV Infection

**Figure 3:**
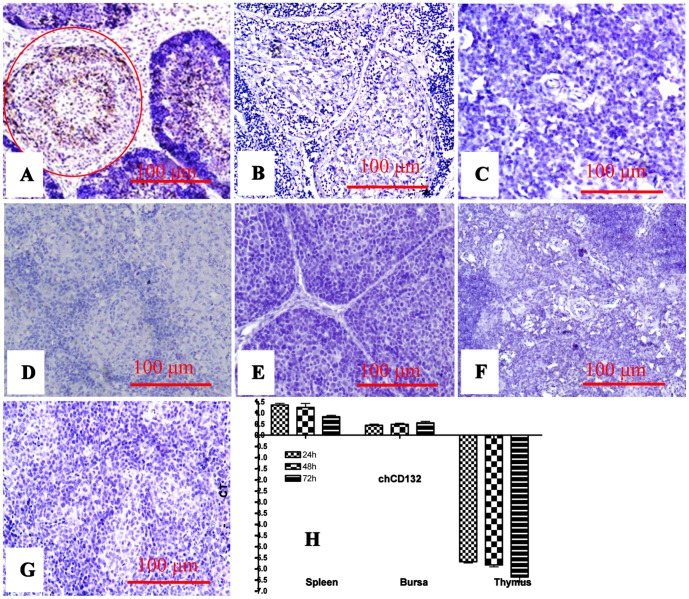
Expression and transcription of chCD132 mRNAs in SPF chickens infected with IBDV. (A)–(G) represents immunohistochemical staining of bursa, thymus, and spleen of mock- and IBDV-infected chickens. (A) IBDV antigens (brown) in bursa lymph follicles (red, round); (B) Bursa lymph follicles of IBDV-infected chicken unrecognized by anti-chCD132 mAb; (C) Thymus of IBDV-infected chicken unrecognized by anti-chCD132 mAb; (D) ChCD132 antigens in spleen of IBDV-infected chicken unrecognized by anti-chCD132 mAb; (E)–(G) Bursa, thymus and spleen of mock-infected chickens un-reactive with anti-chCD132 mAb; (H) chCD132 mRNA transcription in bursa, thymus, and spleen of chicken infected with IBDV. Samples were normalized with the β-actin gene as a negative control, and uninfected SPF chicken as a reference. All samples were assayed in triplicate and the values are expressed as −ΔΔCT ± SD.

The chCD132 was analysed in bursa of chickens infected with virulent IBDV. As shown in Fig. 3A–G, an immunohistochemical assay demonstrated that chCD132 protein is not detected with mouse anti-chCD132 mAb at different time points when post-inoculated in the bursa, thymus, and spleen of mock- and IBDV-infected chickens. Correspondingly, when compared to the mock-infected chickens, up-regulation of chCD132 was not significant (p>0.05) in spleen and bursa of IBDV-infected chickens (Fig. 3H). However, down-regulation of chCD132 mRNA transcription within thymus of IBDV-infected chickens was down regulated. These data indicate that the change of chCD132 transcription within the bursa is insignificant during IBDV infection and that chCD132 transcript is gradually decreased in the spleen and inhibited in thymus.

### Transcriptional Kinetics of γc Cytokines in IBDV-infected Chickens

**Figure 4:**
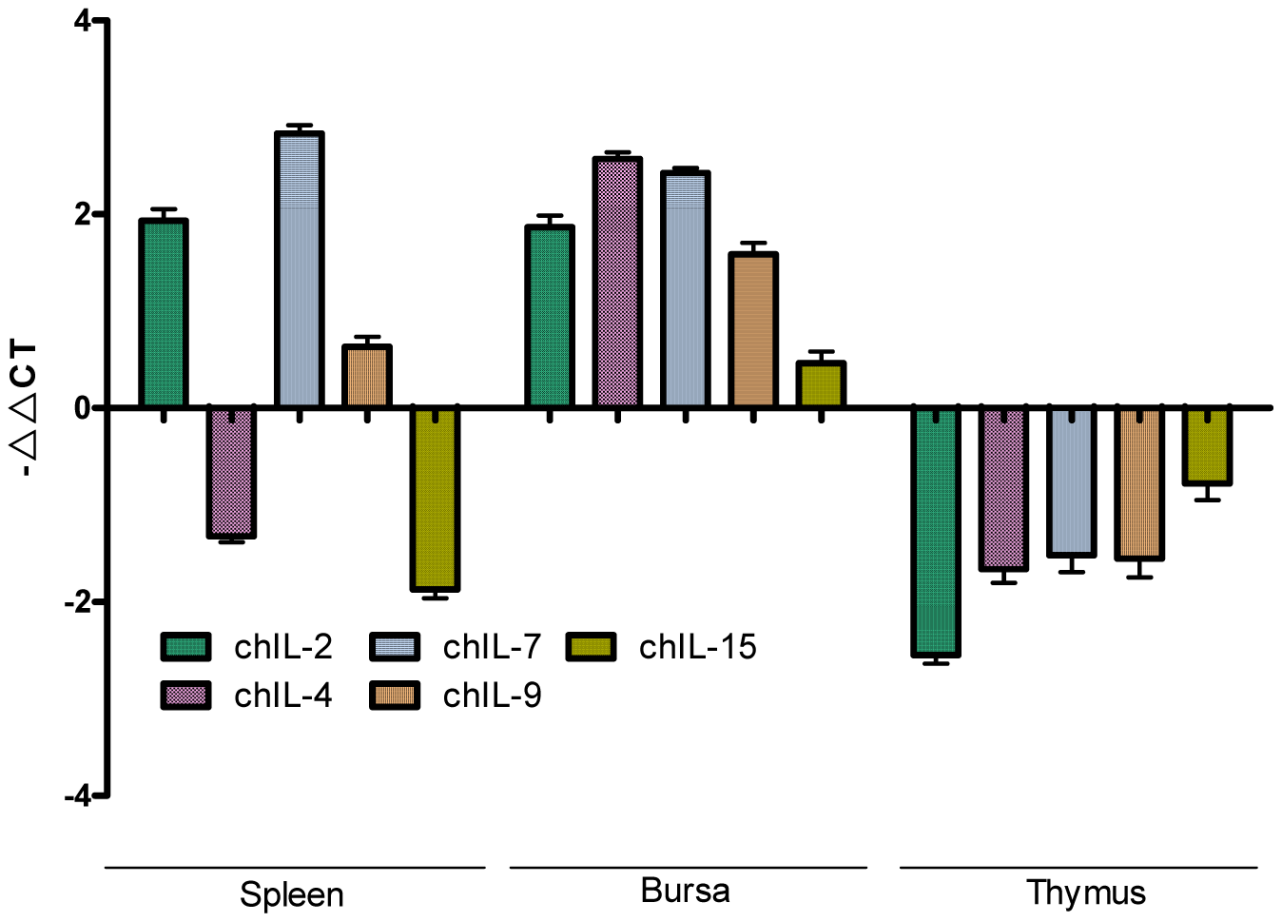
Kinetics of γ_c_ cytokines mRNA expression in chicken immune tissues during infection measured by qRT-PCR. Samples from spleen, thymus, and bursa were normalized with the β-actin gene as a control. All cDNAs were assayed in triplicate. The values are expressed as −ΔΔCT ± SD.

Data shown in Fig. 4 demonstrates that IBDV infection has opposite effects on the transcription of thymic and bursal γ_c_ cytokines in chickens, that is, the transcription of all 5 γ_c_ cytokines is downregulated in thymus and upregulated in bursa. However, in the spleen of IBDV-infected chickens, the transcription of chIL-4 and chIL-15 is downregulated, and the transcription of chIL-2, chIL-7 and chIL-9 is upregulated in comparison to mock infected chickens. This indicates that the balance of γ_c_ cytokines changed in spleen of IBDV-infected chickens.

### Kinetics of CD132-phenotypic T Cells of Chickens Infected with the Virulent IBDV

**Figure 5:**
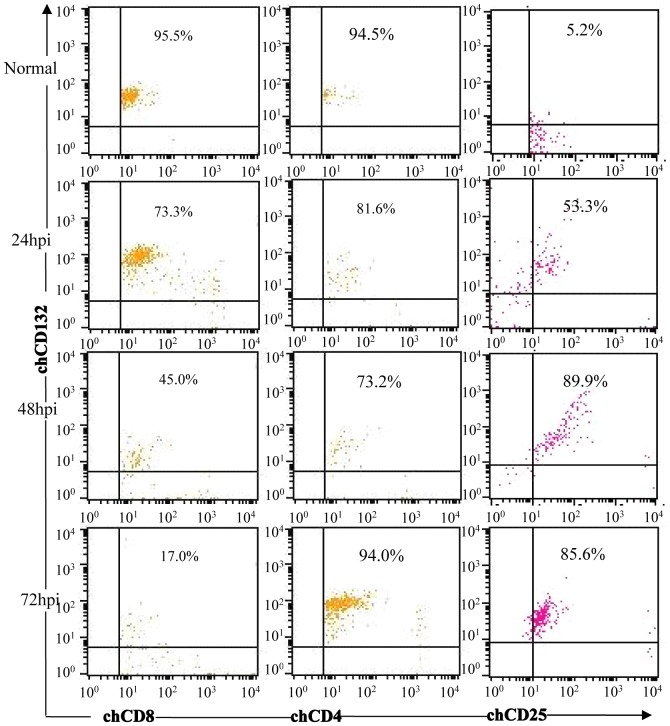
FACS analysis of chCD132-phenotypic T lymphocytes in bursa of IBDV-infected chickens. Bursacytes were incubated with FITC-labeled mouse anti-chicken CD3 mAb and PE-labeled mouse anti-chicken CD4 or CD8 mAb and APC-labeled mouse anti-chicken CD132 mAbs. Cells were counted on a Cytomics FC500 and analyzed using CELLQUEST software. Mock-infected SPF chickens were used as a negative control. All samples were assayed in triplicate.

To analyze the role of chCD132^+^ T lymphocytes, we measured the profiles of CD132-labeled T lymphocytes in the bursa of IBDV-infected chickens by using a FACS assay after all the detected cell samples were stained using the anti-chCD3 mAb. As shown in Fig. 5, from 24 h to 72 h after IBDV infection, the percentage of bursal CD8^+^CD132^+^ T lymphocytes decreases persistently, in contrast with CD132^+^CD25^+^ T cells in IBDV-infected chickens. However, compared to mock-infected chickens, there are no remarkable changes in CD132^+^CD4^+^ T cell frequency (Fig. 5) in bursa of IBDV-infected chickens. These data show that IBDV infection induces downregulation of CD132^+^CD8^+^ T cells and upregulation of CD132^+^CD25^+^ T cells in bursa of IBDV-infected chickens.

## Discussion

Avian cytokines, like their mammalian counterparts, are influential in host immune response to pathogenic infection [5]. CD132, a shared cellular receptor for IL-2, -4, -7, -9, -15, and -21, plays important roles in immunoregulatory networks. CD132 genetic mutations eliminate the activity of all CD132-dependent cytokines, and which result in human XSCID [6]. However, little information is available regarding CD132 molecules in domestic fowl except for that the *γ_c_ chain* gene and chCD132 functional domain binding to chIL-2 in chickens were identified [4]. In this study, we first developed mouse mAbs to rchCD132 by using rchCD132 expressed in *E. coli* as an immunogen. Subsequently, through chCD132 detection of the Con A-stimulated SMC surface, we obtained one hybridoma cell line secreting an anti-chCD132 mAb that binds cellular chCD132. The ability to produce this interaction provides a crucial tool for future functional investigation of chCD132.

Chickens infected with IBDV experience suppression in both humoral and cellular immunity. To date, little is known about the chCD132 molecules that plays important roles in T and NK cell development and activation of γ_c_ cytokine signal pathways. In present study, the transcription of intrathymic γ_c_ is significantly inhibited even if the intrasplenic and intra-bursal γ_c_ transcripts were slightly upregulated in IBDV-infected chickens (Fig. 3H), demonstrating that IBDV infection influences on γ_c_ transcription. Previous researches reported that the acute phase of IBD lasts for about 7 to 10 days; within this phase, the thymus undergoes marked atrophy and extensive apoptosis of thymocytes and its lesions are quickly overcome [7]. Therefore, we guessed that the possible explanation could be that γ_c_-labeled cells impaired in thymus resulted in downregulation of γ_c_ transcripts and that γ_c_-labeled cells activated in spleen and bursa revealed upregulation of γ_c_ transcripts, or that γ_c_-labeled T cells moving from thymus to spleen and bursa induced the downregulation of intrathymic γ_c_ transcripts and upregulation of intrasplenic and intra-bursal γ_c_ transcripts to resist on IBDV infection. However, an accurate explanation regulating γ_c_ transcription needs further investigation during IBDV infection.

The γ_c_ family cytokines broadly contribute to lymphocyte proliferation, differentiation and survival [3]. IL-2 promotes proliferation of B cells, contributes to the development of regulatory T cells and regulates the proliferation and apoptosis of activated T cells [8]. IL-4 is required for T helper 2 cell developments and is a potential contributor to impaired CD8^+^ T-cell function in some anti-viral responses [9]. IL-7 has a central role in T-cell development [10], [11], [12], increased intrathymic IL-7 signaling significantly enhances the maintenance of immature thymocytes. IL-9 induces activation of epithelial cells, B cells, eosinophils, and mast cells [13]. IL-15 has stimulatory activity for the induction of B cell proliferation and differentiation [14], and an essential role in CD8^+^ T-cell homeostasis [15]. Our experiments show that in IBDV-infected chickens mRNA transcripts of 5 γ_c_ family cytokines are upregulated in bursa and inhibited in thymus, however in spleen IL-2, -7, and -9 transcripts were upregulated and the transcripts of IL-4 and -15 were inhibited, indicating that IBDV infection results in disruption of homeostasis of γ_c_ family cytokines. On the basis of our experimental results, we considered that the development of thymic T lymphocytes secreting γ_c_ family cytokines may reveal a functional disturbance during IBDV infection. Delusively, we can not explain why are the kinetics of splenic γ_c_ family cytokines different from those of thymus and bursa γ_c_ family cytokines.

Regulation of cell surface γ_c_ expression levels is critical for cellular response by γ_c_ cytokines [3]. Generally, little was known about CD132-phenotypic T cells with CD4 or CD8 molecules, although some reports have demonstrated that HIV infection increased CD132 expression in all CD4^+^ T cells [16] and in human CD4^+^CD8^+^ thymocytes lost IL-7 signaling [11]. Interestingly, in present study, we found that CD132^+^CD8^+^ T cells were decreased in the bursa of IBDV-infected chickens and CD132^+^CD25^+^ T cells were upregulated (Fig. 5), that is, the profile of CD132^+^CD8^+^ and CD132^+^CD25^+^ T cells revealed a reverse dynamic change, and the CD132^+^CD4^+^ T cells is an insignificant decrease. Therefore it was reasonable to hypothesize that cellular immunity was relevant to CD132-immunophentypic T cells labelled with CD8 or CD25 molecules during IBDV infection. However, we cannot explain the roles of CD132^+^CD8^+^ T cells and CD132^+^CD25^+^ T cells during viral infection. Therefore further studies of CD132^+^ T cells are needed in future.

## Materials and Methods

### Viruses and Specific Pathogen-free Chickens

The chicken embryo fibroblast (CEF)-adapted IBDV strain NB (10^7.3^ TCID_50_/0.1 ml, eNB virus), BF-replicated virulent IBDV strain NB (10^6.15^ BLD_50_/0.1 ml, cNB virus) were identified and stored in our laboratory [17], [18]. Specific pathogen-free (SPF) chickens and embryonated chicken eggs were purchased from Beijing Merial Vital Laboratory Animal Technology Co., Ltd, Beijing, China. The use of all laboratory animals in this study was approved by the scientific ethical committee of Zhejiang University.

### Prokaryotic Expression of chCD132 Protein and Monoclonal Antibody to chCD132

Splenic mononuclear cells (SMCs) were prepared as described previously [19]. An SMC culture was grown in RPMI 1640 medium supplemented with 10% NCS and 10 µg/ml con A at a final concentration of 5×10^6^ cells/ml and was incubated at 41°C for 21 h. SMCs were washed 3 times with ice-cold PBS, lysed with Trizol reagent (Invitrogen, Carlsbad, CA), and total cellular RNA was extracted. Nucleotide sequences encoding the chCD132 extracellular domain were amplified from cultured SMC total cellular RNA by RT-PCR and the primer pair: 5′-ATGAATTCGCATCCCCCAGCCCCAAAG-3′ (containing the *Eco*RI site), and 5′-CGGTCGACTCACGTGTGGATCCAGAATC-3′ (containing the *Sal*I site). PCR products were digested with the enzymes *Eco*RI and *Sal*I, and inserted into the pET28a expression vector (Novagen, Madison, WI). After sequencing, the recombinant chCD132 (rchCD132) protein was expressed under IPTG induction in the *Escherichia coli* BL21 (DE3) strain and purified using a nickel column under denaturing conditions following the manufacturer's instructions (Qiagen Inc., Valencia, CA). Finally, the protein was analyzed by SDS-PAGE and western blot with the anti-His mAb (Amersham, USA) as described previously [20]. Preparation of the mAb to chCD132 was performed according to a previously reported protocol [19]. The mAb binding to native chCD132 was identified using Con A-activated SMCs in indirect immunofluorescence assays as stated previously [19]; PE-conjugated goat anti-mouse IgG was used as the secondary antibody.

### Virus Inoculation and Sampling

CEFs were prepared from 10-day-old SPF embryonated chicken eggs and maintained in Hank's medium supplemented with 8% NCS, then seeded in 96- or 24-well plates. After formation of a CEF monolayer, the monolayer was inoculated with a 100 TCID_50_ dose of the eNB virus. The media-treated CEF monolayers were used as negative controls. The inoculated CEF monolayer was incubated at 37°C for 24 h, 48 h, and 72 h according to experimental demands, and 3 samples were collected at each time point. SPF chickens (25 days old) were raised in negative pressure isolators and inoculated intranasally at 0.1 ml/per chicken with a 300 BLD_50_ dose of the cNB virus. At 48 h post-infection (hpi), blood, spleen, thymus, and bursa from infected chickens were collected for RNA isolation, fluorescence-activated cell sorting (FACS) analysis, and immunohistochemical staining. The mock-infected SPF chickens were used as a negative control. The tissue samples of 3 infected chickens were collected at each time point.

### Quantitative Real-time RT-PCR (qRT-PCR)

Primers specific for simultaneous amplification of γ_c_ cytokine genes, summarized in Table 1, were designed using the Lasergene sequence analysis software (DNAStar, Inc., Madison, WI) according to available gene information in the GenBank TM library. Total cellular RNA was extracted from the CEF monolayer and chicken tissues by using the Trizol reagent (Invitrogen), and RNA concentrations were detected using a spectrophotometer (260/280 nm). After heating at 65°C for 5 min to denature RNA and inactivate RNases, 1 µg of total RNA was subject to reverse transcription using 200 units of SuperScript III reverse transcriptase (Invitrogen), 40 units of RNaseOUT recombinant RNase inhibitor (Invitrogen), 200 ng of random hexamer primers (TaKaRa), 0.5 mM (each) dNTPs (TaKaRa), 4 µl of 5× First-Strand Buffer (Invitrogen), and 1 µl of 0.1 M DTT (Invitrogen) in a total volume of 20 µl at 25°C for 5 min, and then, incubated at 50°C for 1 h. The reaction was terminated by heating at 70°C for 15 min. qRT-PCR was performed by using 500 Real Time PCR system (ABI) in a total volume of 20 µl containing 1 µl cDNA template, 1× SYBR Premix Ex Taq (Perfect Real Time, TakaRa), and 200 nM of each primer. Melting curves were generated, and quantitative analysis of the data was performed using the realplex 2.0 software in a relative quantification (ddCt) study model (Applied Biosystems). The cellular *β-actin* gene previously used as the reference gene [21] in the IBDV infection was taken in the present study. Each sample was assayed in triplicate. Parallel mock-infected CEFs or chicken tissues were used as negative controls. Relative expression level changes greater than 2-fold (ddCT ≥1, p≤0.05) were considered significant.

**Table 1:** Primers for chicken CD132-dependent cytokines and β-actin in the method of Real-time PCR.

Sequences of primers (5′-3′)	Source	Accession No
TCAGGGTGTGATGGTTGGT	β-actin	L08165
TCTGTTGGCTTTGGGGTT		
ATCTTTGGCTGTATTTCGGTAG	chIL-2	AF502412
CCTGGGTCTCAGTTGGTGTGT		
AGTGCCGCTGATGGAGA	chIL-4	AJ621249
GGAGCTGACGCATGTTGA		
CATACTCAGCCATGACATCGA	chIL-7	AJ852017
AGAACCTGTAACGTCCCACA		
CTCTCTTGTGTGCTGCTCTCTG	chIL-9	DQ294769
CAACATGTGCTTGGTTCCTTC		
ATGCTGGGGATGGCACA	chIL-15	AF152927
GCACATAGGAAGAAGATGGTTAGT		

### Indirect Immunofluorescence Assay

Indirect immunofluorescence assays (IFA) were performed as stated previously [22]. CEFs inoculated with the eNB virus were cultured at 37°C for 24 h, 48 h, and 72 h, respectively. The cells were washed with PBS and fixed with cold acetone/methanol (1∶1) for 20 min at −20°C. After washing, CEFs were respectively incubated with anti-chCD132 mAbs at a 1∶500 dilution, followed by incubation with PE-conjugated goat anti-mouse IgG (1∶1000, Southern Biotechnology Associates Inc., Birmingham, AL) for 1 h at 37°C in a humidified chamber. Subsequently, the stained CEF monolayer was incubated with chicken anti-IBDV serum prepared in our laboratory [23], followed by incubation with FITC-conjugated goat anti-chicken IgG (1∶1000; Sigma Chemical Co., St.Louis, MO). Nuclear staining with 4′,6-diamidino-2-phenylindole (DAPI; Sigma) was performed as described previously [24]. The triple-stained cells were washed 3 times with PBS. Finally, the stained cells were examined under a Zeiss LSM510 laser confocal microscope.

### Immunohistochemical Staining

The levels of chCD132 protein in IBDV-infected chicken tissues were measured to understand the kinetics of *in vivo* protein expression. Immunohistochemical assay (IHC) was performed as previously described [25]. Briefly, the collected immune tissues were fixed in 10% neutral buffered formalin, routinely processed, and embedded in paraffin. Subsequently, the sections were treated with 0.3% H_2_O_2_ in PBS to inactivate endogenous peroxidase, washed 3 times in PBS, and digested for 10 min at 37°C with 0.1% trypsin (pH 7.6, 0.1% CaCl_2_) for antigen retrieval. The mAbs specific for chCD132, chicken anti-IBDV serum was applied and allowed to incubate for 2 h at 37°C. The primary antibody was detected by HRP-conjugated goat anti-mouse IgG or goat anti-chicken IgG secondary antibody (KPL, Gaithersburg, Maryland, USA). Finally, the sections were counterstained with hematoxylin.

### Measurement of chCD132 Expression

Anti-chCD132 and anti-chCD25 mAbs were labeled with allophycocyanin (APC) and peridinin-chlorophyll (PerCP) (Donjindo, Japan, and Prozyme, USA, respectively) according to the labeling kit instructions. The bursa of IBDV-infected chickens described above were minced and passed through a stainless steel screen to obtain homogeneous cell suspensions, and bursacytes from IBDV-infected SPF chicken were isolated with Histopaque-1077 (Sigma). To examine CD132 expression in T lymphocytes, bursacytes were stained with an FITC-labeled mouse anti-chicken CD3 mAb (Southern Biotechnology Associates (SBA), Birmingham, AL), PE-labeled mouse anti-chicken CD4 or CD8 mAb (SBA), PerCP-labeled mouse anti-chicken CD25, and APC-labeled mouse anti-chicken CD132 mAb. Each experiment included a control sample with the same combination of antibodies. FACS analysis was performed on a Cytomics FC500 (Beckman Coulter, USA) and analyzed using CELLQUEST software (Beckman).

### Statistical Analysis

All data analysis was performed using Microsoft Excel 2007. Student's t-test was used to detect significant differences between infected and control groups. A P-value ≤0.05 was considered significant.
